# Supplementary material for: Urban areas as hotspots for bees and pollination but not a panacea for all insects
Source: Nat Commun. 2020 Jan 29;11:576. doi: 10.1038/s41467-020-14496-6 (PMC6989530; doi:10.1038/s41467-020-14496-6)
Supplement: Supplementary file 1 — Supplementary Information [file 41467_2020_14496_MOESM1_ESM.pdf]

SUPPLEMENTARY INFORMATION

Theodorou et al. Urban areas as hotspots for bees and pollination but not a panacea for all insects

Supplementary Figures 3

Supplementary Figure 1. Mean nearest taxon distance (MNTD) at rural *versus* paired urban sites...... 3

Supplementary Figure 2. The proportion of green land and edge density (ecotones) in the 1 km surrounding rural *versus* paired rban sites 4

Supplementary Figure 3. Non-metric multidimensional scaling (NMDS) ordination of (a) overall insect communities and (b) Hymenoptera communities 5

Supplementary Figure 4. Main families sampled (>3% of OTUs per order) for each insect order 6

Supplementary Figure 5. Results from a previous study conducted in 2013 and demonstrating the positive correlations across plant species in seed set of (a) *Borago officinalis*, (b) *Sinapis alba*, (c) *Trifolium pratense* and (d) *Trifolium repens* 7

Supplementary Figure 6. Rarefaction curves showing the level of saturation of OTU richness of insects in pan trap samples.. 8

Supplementary Tables 9

Supplementary Table 1. Total patch flower richness, abundance of flowers and number of inflorescences of co-flowering *Trifolium pratense* plants within a 200 m buffer at urban and rural sites. 9

Supplementary Table 2. Best generalised linear model explaining OTU richness of Hymenoptera, bee, Coleoptera, Diptera, hoverflies and Lepidoptera for urban and paired rural sites. 10

Supplementary Table 3. Identity of, and number of interactions with, our *Trifolium pratense* experimental plants per flower-visitor morphogroup and site. 13

Supplementary Table 4. Table of path coefficients from the best-fit piecewise SEM of the relationships between local flower richness, *Trifolium pratense* seed set, *T. pretense* flower visitation rate, local (patch) and landscape factors and flying insect OTU richness across all 18 rural and urban ecosystems 15

Supplementary Table 5a. Table of path coefficients from the best-fit piecewise SEM of the relationships between local flower richness, *Trifolium pratense* seed set, *T. pretense* flower visitation rate, local (patch) and landscape factors and flying insect OTU richness across the nine rural sites.. 16

Supplementary Table 5b. Table of path coefficients from the best-fit piecewise SEM of the relationships between local flower richness, *Trifolium pratense* seed set, *T. pretense* flower visitation rate, local (patch) and landscape factors and flying insect OTU richness across the nine urban sites.................................... 17

Supplementary Table 6. Proportion of the main land cover classes at urban sites. 18

Supplementary Table 7. Proportion of the main land cover classes at rural sites...................................... 19

Supplementary Table 8. Coordinates of field sites used in our study, sampling dates and weather during *Trifolium pratense* observations and pan trapping of flying insects. 20

Supplementary Table 9. Results from a previous study conducted in 2013 around the city of Halle (Germany) on the pollination of four plant species at nine independent sites across an agricultural-urban landscape gradient........ 22

Supplementary Table 10. Land-cover types provided by land cover data obtained from Geofabrik GmbH and average % cover across all sites (rural and urban) at 1,000 m radius from the site centre........ 23

Supplementary Table 11. Pearson correlation coefficients (r) of the relationship between insect species (OTU) richness for the orders Diptera, Lepidoptera, Coleoptera and Hymenoptera, and landscape diversity at increasing area from the centre of a site........ 24

Supplementary Table 12. Number of reads and OTU richness per site. 25

Supplementary Table 13. Pearson correlation coefficients of the relationship between detected OTU richness, rarefied OTU richness and extrapolated total OTU richness 26

Supplementary Table 14. Number of Sanger sequence-generated OTUs for each mock community and 454-generated OTUs successfully blasted to Sanger OTUs at 97% similarity, using both the original pipeline of Yu et al.[4] and our pipeline 27

Supplementary Methods: 28

Metabarcoding: sample preparation, PCR amplification and 454-pyrosequencing for OTU and species assignment........ 28

Supplementary References........ 33

Supplementary Figures

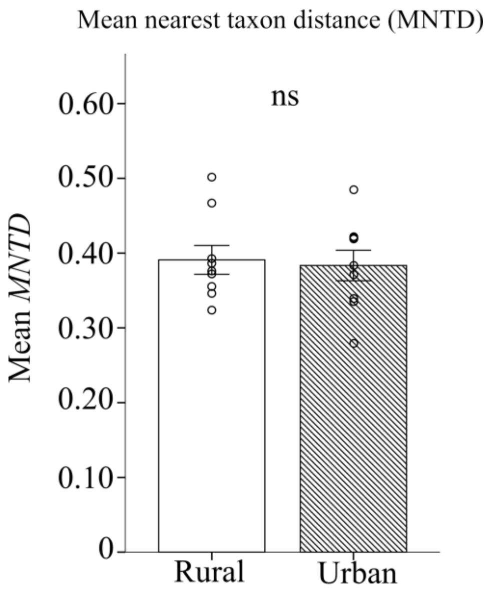

Supplementary Figure 1. Mean nearest taxon distance (MNTD) at N=9 flower-rich rural *versus* N=9 paired flower-rich urban sites. means ± SE are shown; LMM: ns, not significant.

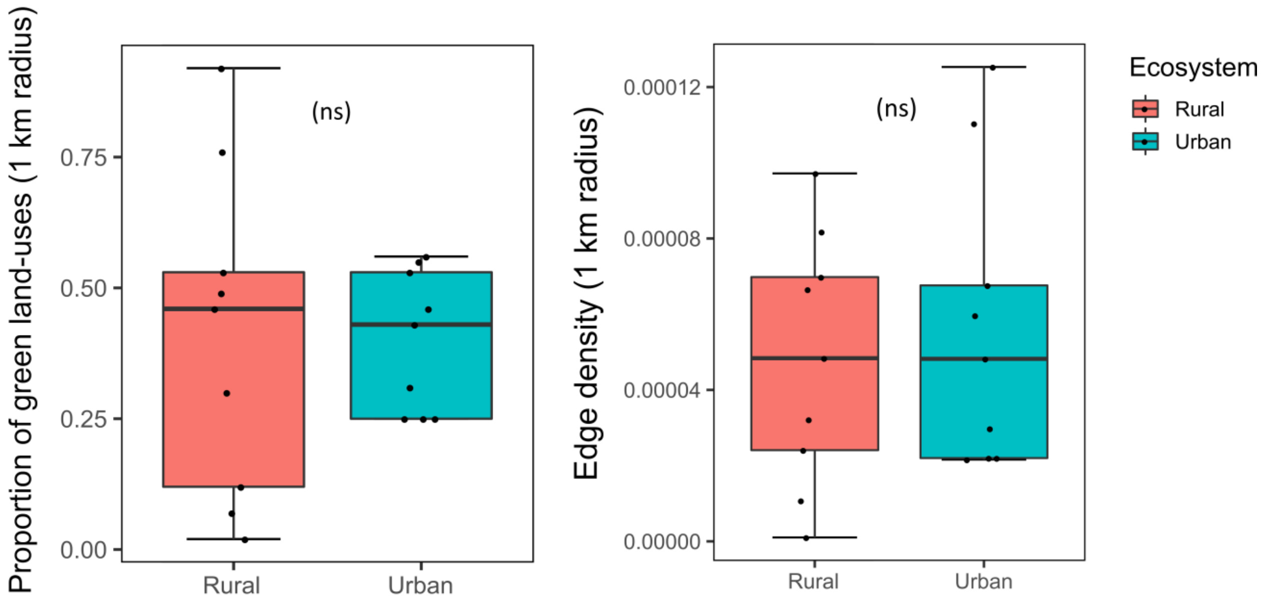

Supplementary Figure 2. The proportion of green land (Forest, semi-natural vegetation, parks and allotments) and edge density (ecotones) in the 1 km surrounding the N=9 flower-rich rural *versus* N=9 paired flower-rich urban sites (green land-uses, LMM, t=-0.080, P=0.938; edge density, LMM, t=0.487, P=0.632). ns, not significant.

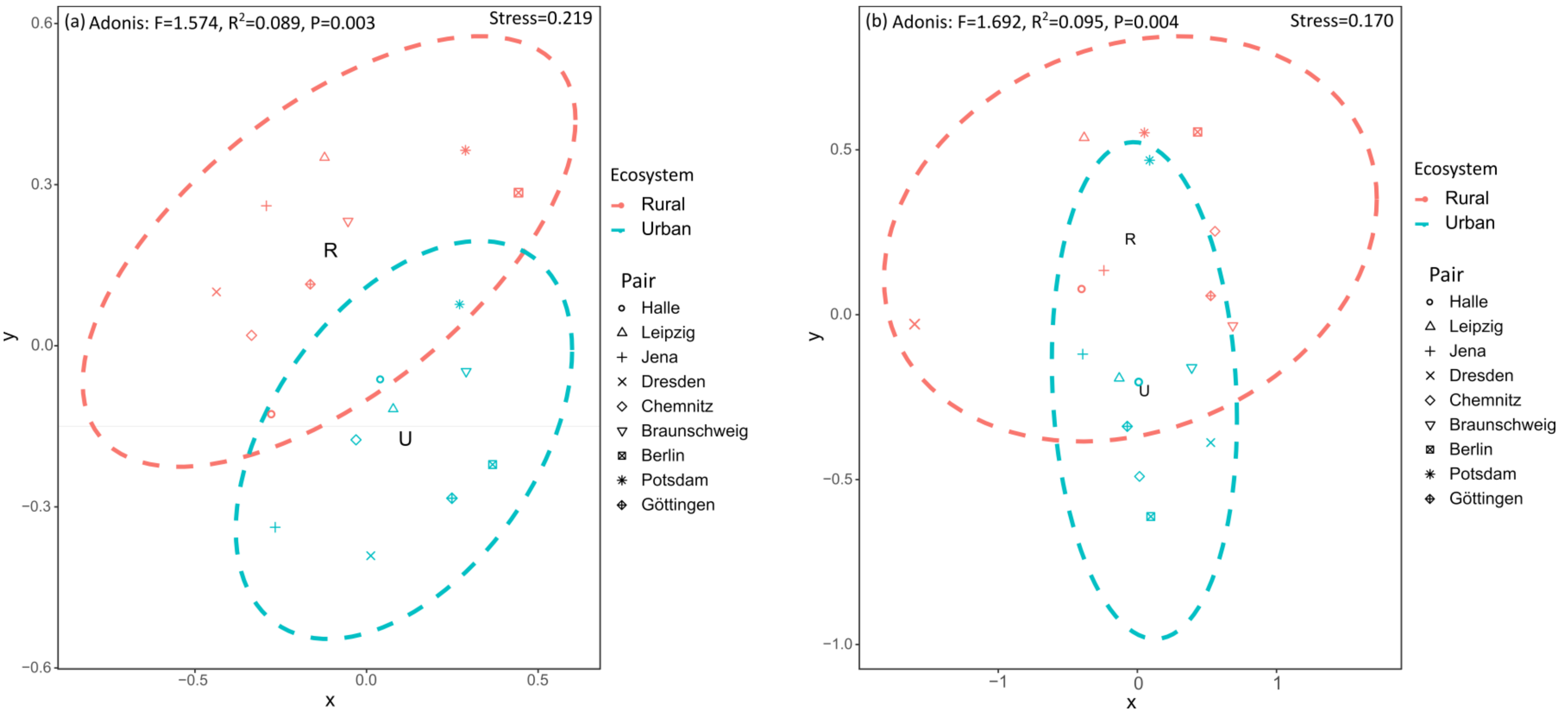

Supplementary Figure 3. Non-metric multidimensional scaling (NMDS) ordination of (a) overall insect communities and (b) Hymenoptera communities. Stress levels are reported in the top right of the figures. Results of the *adonis* analyses of differences in community composition are reported in the top left of the figure.

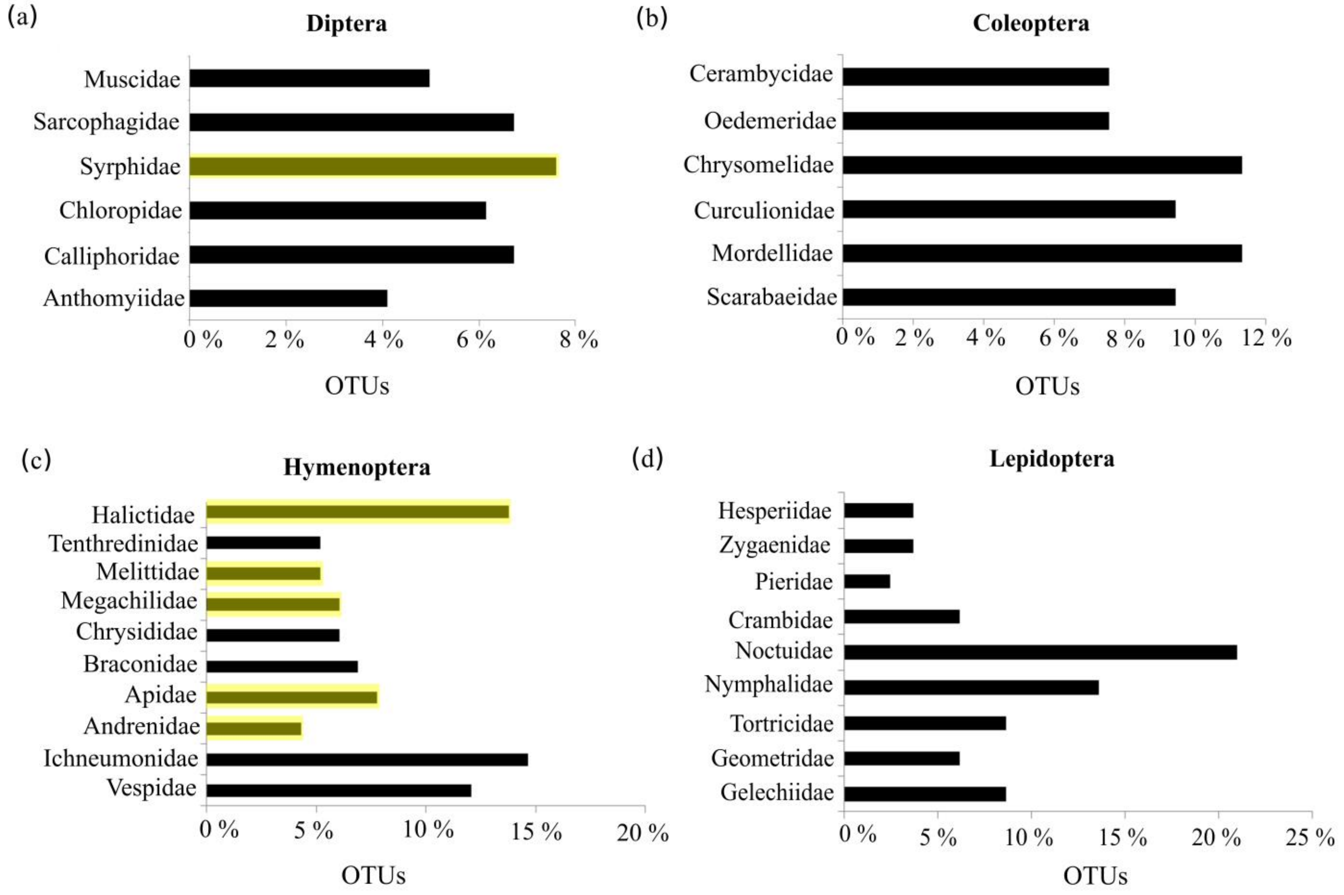

Supplementary Figure 4. Main families sampled (>3% of OTUs per order) for each insect order: (a) Diptera n = 342 OTUs, (b) Coleoptera n = 53 OTUs, (c) Hymenoptera n = 116 OTUs, and (d) Lepidoptera n = 81 OUTs. Highlighted in yellow are the hoverflies (family: Syrphidae) within the Diptera (a), and five families of bee (members of the Anthophila) within the Hymenoptera (c).

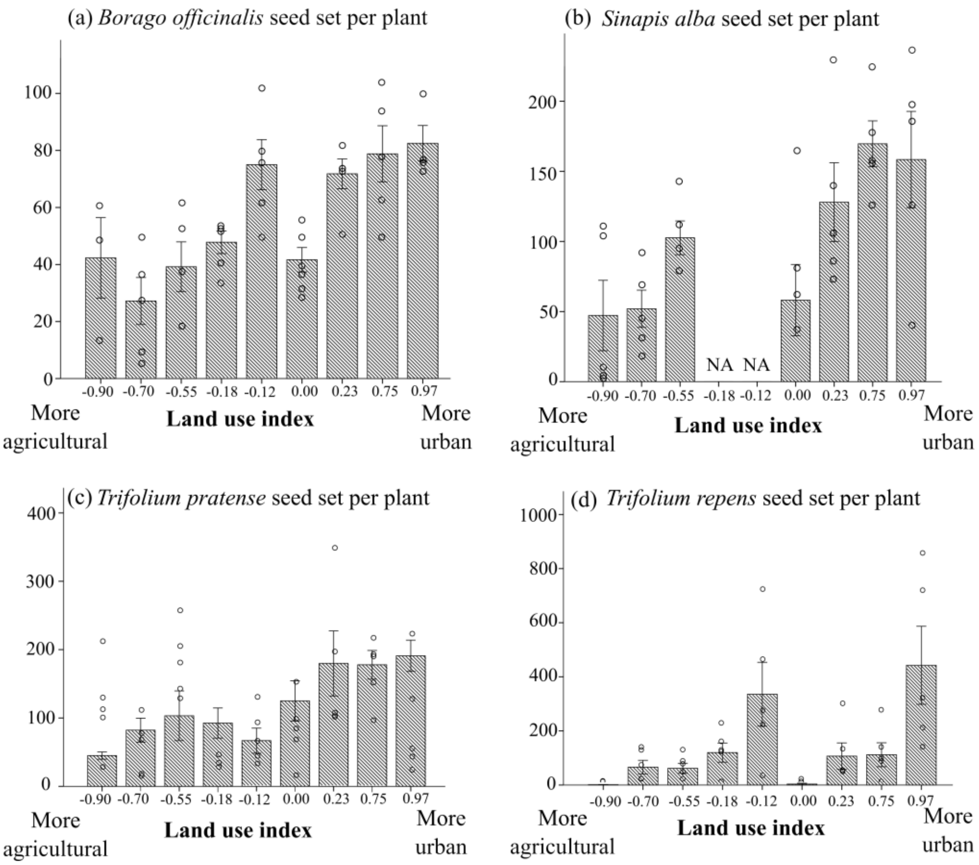

Supplementary Figure 5. Results from a previous study[1,2] conducted in 2013 and demonstrating the positive correlations across plant species in seed set of (a) *Borago officinalis*, (b) *Sinapis alba*, (c) *Trifolium pratense* and (d) *Trifolium repens* across an agricultural-urban landscape gradient around the city of Halle, Germany. The statistical results of the correlations are reported in Supplementary Table 5. Means ± SE as well as individual (overlapping) data points are shown.

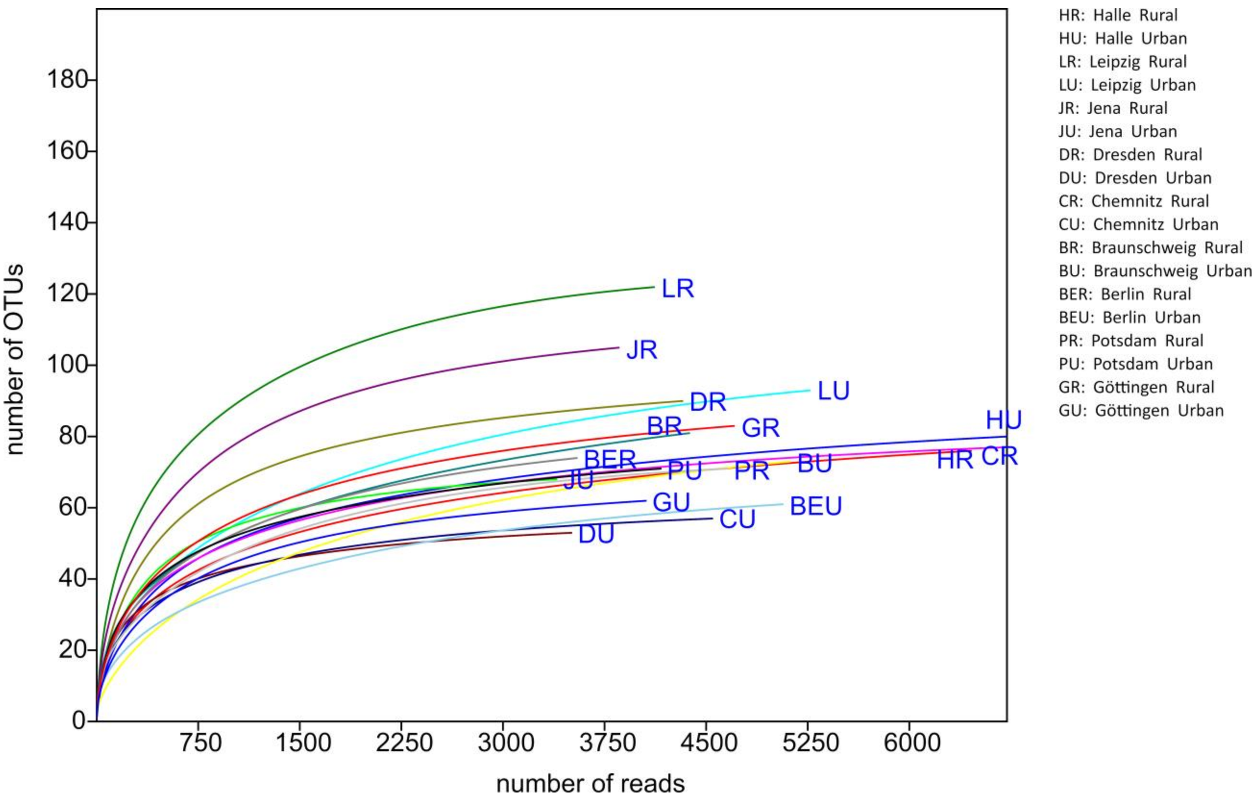

Supplementary Figure 6. Rarefaction curves showing the level of saturation of OTU richness of insects in pan trap samples.

Supplementary Tables

Supplementary Table 1. Total patch flower richness (number of co-flowering plant species), abundance of flowers in 10 x 1 m^2 quadrats, and number of inflorescences of co-flowering *Trifolium pratense* plants within a 200 m buffer at urban and rural sites.

Urban	Flower richness	Flower abundance	No. inflorescences of co-flowering *T. pratense* plants
Halle	13	87.4	3036
Leipzig	13	66.6	68
Jena	22	184.8	1600
Dresden	18	44.1	182
Chemnitz	17	84.3	426
Braunschweig	12	18.2	20
Potsdam	5	30.7	1020
Berlin	12	27.1	340
Göttingen	8	81.7	40
**Average**	13.3 ± 5.1 SD	77.2 ± 46.1 SD	748 ± 1007 SD
**Rural (paired to the like-named city)**			
Halle	7	21.5	1869
Leipzig	16	150.8	376
Jena	10	58.9	1256
Dresden	11	93.7	400
Chemnitz	6	87.5	2100
Braunschweig	6	119.5	1400
Potsdam	5	27.1	55
Berlin	8	123.1	20
Göttingen	4	27.0	500
**Average**	8.1 ± 3.7 SD	73.7 ± 47.9 SD	886 ± 784 SD

Supplementary Table 2. Best generalised linear model (GLM) explaining OTU richness of Hymenoptera, bee (Anthophila, a subset of Hymenoptera), Coleoptera, Diptera, hoverflies (Syrphidae, a subset of Diptera) and Lepidoptera for N=9 urban and N=9 paired rural sites at the local (patch) and landscape scales (1,000 m radius for all Hymenoptera and Coleoptera, 250 m for all Diptera and Lepidoptera). We used AICc for model selection. Due to low sample size (mean=1.44±1.01 SD), Lepidoptera OTU richness was not modelled within urban sites. We did not find any significant predictors of Syrphidae OTU richness in rural or urban ecosystems.

Site type	OTU richness	Parameters	Estimate	SE	Z-value	P-value	R^2_{adj}
Urban							
	Hymenoptera	Intercept	2.679	0.089	29.990		
		Edge density (1,000 m)	0.189	0.074	2.544	0.011*	0.638
	Bee/Anthophila	Intercept	2.143	0.118	18.07		
		Edge density (1,000 m)	0.305	0.096	3.16	0.001**	0.707
	Coleoptera	Intercept	1.677	0.151	11.048		
		Habitat diversity	0.372	0.115	3.225	0.001**	0.771
	Diptera	Intercept	3.725	0.051	71.670		

		Proportion of residential cover (250 m)	0.124	0.057	2.172	0.029*	0.639
		Edge density (250 m)	0.110	0.045	2.436	0.014*	
Rural							
	Hymenoptera	Intercept	2.851	0.130	21.906		
		Edge density (1,000 m)	0.463	0.188	2.530	0.011*	0.770
		Proportion of arable land (1,000 m)	-0.438	0.146	-3.001	0.002**	
	Bee/Anthophila	Intercept	2.087	0.192	10.874		
		Edge density (1,000 m)	0.518	0.209	2.477	0.013*	0.591
		Proportion of arable land (1,000 m)	-0.468	0.221	-2.112	0.034*	
	Coleoptera	Intercept	2.162	0.132	16.383		
		Local flower richness	0.651	0.194	3.352	>0.001***	0.845
		Habitat diversity	0.709	0.287	2.471	0.013*	

Diptera	Intercept	4.037	0.053	75.453		
	Local flower richness	0.159	0.063	2.517	0.011*	0.455
Lepidoptera	Intercept	2.599	0.105	24.637		
	Local flower richness	0.398	0.127	3.128	0.001**	0.640

*, P<0.05; **, P<0.01; R^2_{adj}= Proportion of the variance in the dependent variable that is predicted from the independent variable(s).

Supplementary Table 3. Identity of, and number of interactions with, our *Trifolium pratense* experimental plants per flower-visitor morphogroup and site.

**Site**	**Coleoptera**	**Syrphidae**	**Other Diptera**	**Lepidoptera**	**Wasps**	**Andrenidae**	**Halictidae**	***Bombus lapidarius/ sorooensis proteus***	***Bombus terrestris/lucorum***	***Bombus pascuorum***	***Apis mellifera***
Rural Halle	0	3	2	0	0	3	4	16	0	1	2
Urban Halle	0	1	0	0	2	3	2	0	0	30	3
Rural Leipzig	3	1	0	16	0	1	2	27	2	19	0
Urban Leipzig	0	5	0	0	0	2	3	2	3	107	31
Rural Jena	0	1	6	25	0	0	6	24	1	0	2
Urban Jena	0	2	0	0	0	0	4	2	9	26	4
Rural Dresden	2	1	7	3	0	7	21	0	26	9	1
Urban Dresden	0	1	0	0	0	2	5	0	22	131	22
Rural Chemnitz	1	2	1	7	0	2	6	7	18	0	1
Urban Chemnitz	0	2	0	4	0	0	0	0	0	153	11
Rural Braunschweig	0	2	0	1	0	0	6	5	16	10	1
Urban Braunschweig	0	1	0	1	0	8	2	0	11	59	4
Rural Berlin	0	0	0	0	0	0	0	2	4	10	0
Urban Berlin	0	1	0	0	0	0	0	2	0	42	2

Rural Potsdam	0	0	1	3	0	0	0	4	51	1	0
Urban Potsdam	0	0	0	0	0	3	3	0	0	70	0
Rural Göttingen	0	0	0	0	0	0	0	0	16	0	17
Urban Göttingen	0	4	0	0	0	0	1	0	0	46	13
TOTAL	6	27	17	60	2	31	65	91	179	714	114

Supplementary Table 4. Table of path coefficients from the best-fit piecewise SEM of the relationships between local flower richness, *Trifolium pratense* seed set, *T. pretense* flower visitation rate, local (patch) and landscape factors and flying insect OTU richness across all 18 rural and urban ecosystems (visualization: Figure 7 in the main text). The SEM showed stable fit to our data (Fisher's C=8.03, d.f.=4, P=0.09).

Response		Predictor	Estimate (Unstandardized)	S.E.	P
Hymenoptera OTU richness	←	Proportion of arable land	-0.262	0.030	<0.001
Hymenoptera OTU richness	←	Edge density	0.098	0.024	<0.001
Visitation rate	←	Proportion of arable land	-6.542	0.877	<0.001
Visitation rate	←	Edge density	3.995	0.628	<0.001
T. pratense seed set	←	Hymenoptera OTU richness	11.743	3.476	0.014
T. pratense seed set	←	Hymenoptera PSV	56.242	15.669	0.015
T. pratense seed set	←	Visitation rate	7.565	1.559	<0.001

S.E. = standard error; P = statistical significance; Hymenoptera PSV = Hymenoptera phylogenetic species variability

Supplementary Table 5a. Table of path coefficients from the best-fit piecewise SEM of the relationships between local flower richness, *Trifolium pratense* seed set, *T. pretense* flower visitation rate, local (patch) and landscape factors and flying insect OTU richness across the nine rural sites. The SEM showed stable fit to our data (Fisher's C=7.096, d.f.=6, P=0.312).

Response		Predictor	Estimate (Unstandardized)	S.E.	P
Hymenoptera OTU richness	←	Edge density	0.414	0.054	<0.001
Hymenoptera OTU richness	←	Proportion of arable land	-0.517	0.052	<0.001
T. pratense seed set	←	Hymenoptera PSV	53.166	21.226	0.05
T. pratense seed set	←	Hymenoptera OTU richness	8.167	2.657	0.027
T. pratense seed set	←	Visitation rate	11.337	3.298	<0.001

S.E. = standard error; P = statistical significance; Hymenoptera PSV = Hymenoptera phylogenetic species variability

Supplementary Table 5b. Table of path coefficients from the best-fit piecewise SEM of the relationships between local flower richness, *Trifolium pratense* seed set, *T. pretense* flower visitation rate, local (patch) and landscape factors and flying insect OTU richness across the nine urban sites. The SEM showed stable fit to our data (Fisher's C=7.712, d.f.=7, P=0.462).

Response		Predictor	Estimate (Unstandardized)	S.E.	P
Hymenoptera OTU richness	←	Edge density	0.186	0.020	<0.001
Visitation rate	←	Edge density	4.333	1.895	0.050
T. pratense seed set	←	Hymenoptera PSV	45.737	20.131	0.062
T. pratense seed set	←	Hymenoptera OTU richness	9.579	4.271	0.061
T. pratense seed set	←	Visitation rate	6.447	1.817	<0.001

S.E. = standard error; P = statistical significance; Hymenoptera PSV = Hymenoptera phylogenetic species variability

Supplementary Table 6. Proportion of the main land cover classes at urban sites.

City	Proportion of each land cover class (arable, semi-natural, forest and four forms of urban land cover) at 1,000 m radius from the site centre									
	Arable	Semi-natural	Forest (D=deciduous;; M=Mixed)	Residential (domestic housing with gardens)	Commercial/ industrial	Botanical park	Public park	Allotments	Road density (km)	Buildings cover (km^2)
Halle	0	0	0.01 (D)	0.63	0.10	0.20	0.04	0	55.40417	0.713536
Leipzig	0	0.15	0	0.52	0.02	0.14	0.12	0.05	92.65159	0.580860
Jena	0	0.02	0.04 (D)	0.39	0.17	0.31	0.06	0	87.55788	0.716511
Dresden	0	0.08	0.18 (D)	0.44	0.02	0.04	0.23	0	99.73007	0.312379
Chemnitz	0	0	0.20 (M)	0.42	0	0.24	0.06	0.06	55.70457	0.328523
Braunschweig	0	0	0	0.64	0.05	0.25	0.06	0	97.63848	0.878783
Potsdam	0.04	0	0.22 (D)	0.45	0	0.04	0.24	0.05	63.64427	0.150954
Berlin	0.03	0	0.01 (D)	0.68	0.02	0.20	0.04	0	62.52504	0.561380
Göttingen	0	0.07	0	0.72	0.02	0.14	0.04	0	114.73180	0.946886
**Average**	0.01	0.03	0.07	0.54	0.04	0.17	0.10	0.02	78.73	0.58

Supplementary Table 7. Proportion of the main land cover classes at rural sites.

Paired city name of rural site	Proportion of each land cover class (arable, semi-natural, forest and two forms of urban land cover) at 1,000 m radius from the site centre							
	Arable	Semi-natural	Forest (D=deciduous; C=Coniferous))	Residential (domestic housing with gardens)	Commercial/ industrial	Allotments	Road density (km)	Bulidings cover (km^2)
Halle	0.53	0.07	0.03 (D)	0.34	0	0.02	25.39409	0.100467
Leipzig	0	0	0.76 (D)	0.14	0.09	0	21.51763	0.055687
Jena	0.59	0.06	0.24 (D)	0.10	0	0	7.99099	0
Dresden	0.42	0.04	0.49 (C)	0.04	0	0	15.13525	0.018657
Chemnitz	0.62	0.05	0.02 (C)	0.22	0.09	0	12.90439	0.034797
Braunschweig	0.52	0.02	0.44 (D)	0.02	0	0	17.41563	0.004461
Potsdam	0.41	0	0.49 (C)	0.07	0.01	0	25.05485	0.038421
Berlin	0	0.44	0.48 (C)	0.01	0.05	0	17.86504	0.096161
Göttingen	0.95	0	0.02 (D)	0.02	0	0	11.52529	0.011836
**Average**	0.45	0.09	0.33	0.10	0.02	0	17.20	0.04

Supplementary Table 8. Coordinates of field sites used in our study, sampling dates and weather during *Trifolium pratense* observations and pan trapping of flying insects.

Site	Latitude (N)	Longitude (E)	Sampling dates (day/month) in 2014	Weather (morning/afternoon)			Hours of sunshine across 5 days of sampling (h)
				Temperature (°C)	Humidity (%)	Wind (ms^{-1})	
Rural Halle	51.39112	11.87891	12/06-17/06	17.7/20.1	47.8/38.1	1.8/0.9	34
Urban Halle	51.48966	11.96135	12/06-17/06	25.4/23.6	40.8/47.3	0.9/1.2	34
Rural Leipzig	51.18594	12.49890	26/06-01/07	25.7/29.2	48.6/39.8	0.4/0.3	35
Urban Leipzig	51.32920	12.39198	26/06-01/07	18.9/25.7	62.8/41.8	0.4/0.7	35
Rural Jena	50.82824	11.30146	26/06-01/07	18.0/19.5	40.0/37.1	1.8/1.5	35
Urban Jena	50.93119	11.58430	26/06-01/07	21.4 /23.0	44.8/36.8	0.5/0.4	35
Rural Dresden	50.94165	13.43837	02/07-06/07	23.9/28.9	35.3/29.6	1.5/1.6	34
Urban Dresden	51.04314	13.75754	02/07-06/07	22.9/29.3	41.1/28.0	0.7/1.1	34
Rural Chemnitz	50.96313	13.08918	02/07-06/07	28.3/27.1	38.6/28.0	0.9/0.8	34
Urban Chemnitz	50.85040	12.89103	02/07-06/07	23.5/26.2	45.6/34.4	0.4/0.6	34
Rural Braunschweig	52.20853	11.11153	15/07-19/07	25.0/28.0	55.2/42.2	0.4/0.4	36

Urban Braunschweig	52.26870	10.53336	15/07-19/07	23.1/25.0	60.7/51.1	0.3/0.3	36
Rural Berlin	52.16986	13.48448	31/07-03/08	25.6/26.0	61.3/45.0	0.8/1.8	32
Urban Berlin	52.45289	13.31002	31/07-03/08	25.0/29.0	58.0/36.0	1.6/1.9	32
Rural Potsdam	52.28192	12.83659	31/07-03/08	21.0/30.0	62.0/53.0	0.1/0.8	32
Urban Potsdam	52.40796	13.02213	31/07-03/08	27.0/27.0	48.0/39.0	0.3/1.0	32
Rural Göttingen	51.54377	10.38625	6/08-10/08	25.0/27.0	59.0/52.0	0.8/0.4	31
Urban Göttingen	51.53826	09.93850	6/08-10/08	22.0/29.0	60.0/42.5	0.4/0.8	31

Supplementary Table 9. Results from a previous study[1,2] conducted in 2013 around the city of Halle (Germany) on the pollination of four plant species at nine independent sites across an agricultural-urban landscape gradient. Pearson correlation coefficients (*r*, below diagonal) of the relationship between seed set of *Borago officinalis, Sinapis alba*, *Trifolium pratense* and *Trifolium repens* and significance (uncorrected *P* values, above diagonal). There was a positive correlation in seed set for each pair of plant species across the nine sites except *B. officinalis*-*T. repens*, for which the relationship was non-significant (r=0.688, P=0.119).

	Borago officinalis	*Sinapis alba*	*Trifolium pratense*	*Trifolium repens*
Borago officinalis	-	0.004	0.027	0.119
Sinapis alba	0.914	-	0.008	0.013
Trifolium pratense	0.808	0.955	-	0.013
Trifolium repens	0.688	0.645	0.680	-

Supplementary Table 10. Land-cover types provided by land cover data obtained from Geofabrik GmbH and average % cover across all sites (rural and urban) at 1,000 m radius from the site centre. We split the Geofabrik feature class 'park' into two for our analyses based on ground truthing: botanical park and public park; we also combined four Geofabrik feature classes into one for our analyses: meadow, nature reserve, grass and scrub were combined into semi-natural.

Geofabrik feature class	Description	Classes used in analyses	% cover at 1,000 m
Forest	A forest or woodland	Forest	20.16%
Park	A park	Botanical park	8.66 %
		Public park	4.94%
Residential	A residential area	Residential	32.50%
Industrial	An industrial area	Commercial/Industrial	3.55%
Arable land	Agricultural land (farms and areas where crops are grown)	Arable (= agricultural)	22.83%
Allotments	An area with small private gardens	Allotments	1.00%
Meadow	A meadow, possibly used for grazing cattle	Semi-natural	1.79%
Nature reserve	A nature reserve	Semi-natural	0.33%
Quarry	A quarry	Quarry	0.09%
Grass	Semi-natural grassland	Semi-natural	2.78%
Scrub	Area of scrub vegetation	Semi-natural	0.59%

Supplementary Table 11. Pearson correlation coefficients (r) of the relationship between insect species (OTU) richness for the orders Diptera, Lepidoptera, Coleoptera and Hymenoptera, and landscape diversity (measured as Shannon-Weiner diversity of land-uses) at increasing area (given as radius in metres) from the centre of a site. The largest absolute correlation coefficient is given in bold.

Radius	250 m	500 m	750 m	1,000 m	1,500 m
Combined rural and urban					
All Insecta OTU richness	**-0.59**	-0.31	-0.18	-0.09	0.01
Diptera OTU richness	**-0.44**	-0.32	-0.32	-0.27	-0.23
Lepidoptera OTU richness	**-0.67**	-0.39	-0.33	-0.28	-0.15
Coleoptera OTU richness	0.04	0.09	0.28	**0.33**	0.15
Hymenoptera OTU richness	0.00	0.18	0.30	**0.38**	0.33
Rural					
All Insecta OTU richness	-0.10	**-0.35**	-0.19	-0.22	-0.31
Diptera OTU richness	**-0.28**	-0.25	-0.19	-0.16	-0.20
Lepidoptera OTU richness	**-0.55**	-0.25	-0.24	-0.29	-0.38
Coleoptera OTU richness	0.21	0.02	0.20	**0.36**	0.24
Hymenoptera OTU richness	-0.08	-0.11	-0.01	**-0.19**	-0.07
Urban					
All Insecta OTU richness	**-0.64**	0.04	0.13	0.26	0.24
Diptera OTU richness	**-0.51**	-0.16	-0.23	-0.19	-0.28
Lepidoptera OTU richness	**-0.69**	-0.13	0-.41	-0.21	0.01
Coleoptera OTU richness	0.26	0.37	0.62	**0.80**	0.69
Hymenoptera OTU richness	0.40	0.21	0.31	**0.50**	0.49

Supplementary Table 12. Number of reads and OTU richness per site.

Site	Number of reads	OTU richness
Rural Halle	6475	76
Urban Halle	6732	80
Rural Leipzig	4139	122
Urban Leipzig	5282	93
Rural Jena	3877	105
Urban Jena	3417	68
Rural Dresden	4344	90
Urban Dresden	3528	53
Rural Chemnitz	6729	77
Urban Chemnitz	4569	57
Rural Braunschweig	4392	81
Urban Braunschweig	5133	73
Rural Berlin	3561	74
Urban Berlin	5080	61
Rural Potsdam	4670	71
Urban Potsdam	4183	71
Rural Göttingen	4723	83
Urban Göttingen	4077	62
Total	8 4911	

Supplementary Table 13. Pearson correlation coefficients (*r*, below diagonal) of the relationship between detected OTU richness, rarefied OTU richness and extrapolated total OTU richness (Chao 1) and significance (uncorrected *P* values, above diagonal).

	Detected OTU richness	Rarefied OTU richness	Chao1
Detected OTU richness	1	<0.001	<0.001
Rarefied OTU richness	0.980	1	<0.001
Chao1	0.983	0.949	1

Supplementary Table 14. Number of Sanger sequence-generated OTUs (Sanger OTUs) for each mock community and 454-generated OTUs successfully blasted to Sanger OTUs at 97% similarity, using both the original pipeline of Yu et al.[4] and our pipeline, as well as OTUs we detected with our pipeline that did not match those of Yu et al.[10].

Mock communities	Sanger OTUs	≥1-read OTUs Yu et al. 2012	≥1-read OTUs this study	OTUs that did not match the reference
1H1X	159	107 (67.3%)	129 (81.1%)	13
XSBN	230	156 (67.8%)	168 (73.0%)	7
KMG	152	127 (83.5%)	133 (87.5%)	11
HongHe	167	133 (79.6%)	147 (88.0%)	12
2H1K	140	117 (83.5%)	129 (92.1%)	13
2K1X	134	90 (67.1%)	103 (76.8%)	11
5K1X	106	67 (63.2%)	75 (70.7%)	5
All communities	547	408 (74.5%)	484 (88.5%)	

Supplementary Methods:

Metabarcoding: sample preparation, PCR amplification and 454-pyrosequencing for OTU and species assignment

To generate mitochondrial DNA (*cytochrome oxidase I*) sequences for the identification of flying insect OTUs (our proxy for species) whilst avoiding biases in DNA extraction and PCRs, we used standard methods recommended for insect barcoding (http://ccdb.ca/resources/)[3], as adapted for metabarcoding[4–6]. Insect samples from each site were washed, dried and weighed and, per 10 g of biological material, we added 5 ml of sterile ddH_2O. The entire insect sample per site was then homogenized using a semi-automated Homex6 homogenizer (Bioreba AG, Reinach, Switzerland), after which 15% of the solution was used for genomic DNA extraction with a tissue DNA maxi kit (Omega Bio-tek, Norcross, USA). This gave us one DNA extract (DNA soup) per study site.

The quantity and quality of purified DNA was assessed using an Epoch microplate spectrophotometer (BioTek, Winooski, USA); all samples contained > 100 ng/µL DNA of high purity (A_{260}/A_{280} = 1.7-2.0 for all 18 samples). Each sample was PCR amplified with universal primers LCO-1490 (5'-GGTCAACAAATCATAAAGATATTGG-3') and HCO-2198 (5'-TAAACTTCAGGG-TGACCAAAAAATCA-3')[7] that target the approximately 650 bp 'barcode' region of the mitochondrial cytochrome *c* oxidase subunit I (COI) and that have been successfully used to amplify the barcode region of German insects[8]. The standard Roche A-adaptor and a unique 10 bp MID (Multiplex IDentifier) tag for each sample were attached to the LCO primer. Each sample was amplified in three independent reactions to reduce PCR bias;

PCR products were then pooled per site. PCR reactions were performed in 20 µL volumes consisting of 2X Promega PCR buffer with 3.0 mM $MgCl_2$ (Promega, Fitchburg, USA), 0.4 µM of each primer, 0.2 mM dNTP, 0.5 U GoTaq Polymerase (Promega, Fitchburg, USA) and 60 ng of template DNA. We performed PCRs for each site separately to avoid cross contamination. Within each PCR reaction, a negative control lacking DNA template was always included to detect contamination from extraneous sources such as PCR reagents; contamination was never detected. PCRs were performed with a Biometra TProfessional basic gradient thermocycler (Biometra, Göttingen, Germany) using the following thermal cycling program: initial denaturation at 94°C for 3 min, followed by 35 cycles of 94°C for 30 s, annealing temperature at 51°C for 45 s and 72°C for 1 min, plus a final extension step of 72°C for 8 min. We have found these PCR criteria (master mixes of PCR components, cycling temperatures and durations) to allow successful amplification of the German bee fauna[9]. PCR products were quantified and visualized through a QIAxcel automated capillary electrophoresis system (Qiagen, Hilden, Germany). No signal was visible in the negative (no DNA template) control for the PCR reactions whereas a single, clear product of ca. 650 bp was visualised in all samples.

For 454-pyrosequencing, all PCR products were purified using a QIAquick PCR purification kit (Qiagen, Hilden, Germany), quantified using the Quant-iT PicoGreen dsDNA Assay kit (Invitrogen, Grand Island, USA) and diluted down to $1x10^9$ molecules/µl. Pooled and labelled PCR products were sequenced on a 454/Roche GS-FLX Plus System (Macrogen, Seoul, Korea) next generation sequencing (NGS) machine.

DNA concentrations at each measurement step (pre- and during NGS library preparation) were consistent across samples. For example, we used 60 ng of template DNA in all PCR reactions to

amplify the COI region and all purified PCR products were diluted down to $1x10^9$ molecules/µl prior to NGS sequencing. Potential taxon-specific PCR amplification biases should therefore have been consistent across all samples and not have influenced downstream bioinformatics or statistical analyses.

Bioinformatics analysis of metabarcoding data

We employed well-established and robust software and pipelines for OTU assignment using metabarcode data[10–15] that we independently verified and that gave consistent OTU and species assignments when altering parameter settings of bioinformatics algorithms.

Step 1: *quality filtering.* Low quality reads were removed using a strict quality filter applied with FlowClus 1.1[10] and cutadapt 1.14[11]. Reads were retained if they (i) matched one of the MID tags with one mismatch allowed, (ii) contained the forward primer with 4 mismatches allowed, (iii) were at least 395 bp long, (iv) had at least a mean Phred score of 30 on the trimmed length, (v) did not hold any ambiguous nucleotides, (vi) had homopolymers no longer than 12 bp, and (vii) had a flowgram length of at least 360, as previously advised for 454 GS FLX reads[12]. The reads were subsequently denoised using FlowClus, which has been shown to recover sequences with a lower error rate and be more easily applicable to large sequence datasets than the original 454 denoising algorithm AmpliconNoise[10,12]. The denoised and quality filtered reads were trimmed to their first 395 bp and potential chimeras were removed using UCHIME 4.2.40[13], as implemented in MOTHUR[14].

Step 2: de novo *clustering and taxonomic assignment.* Quality filtered reads were de-replicated, sorted in decreasing order of abundance and clustered into OTUs with a global threshold of 97% similarity using the cd-hit-est program[15]. The most abundant read in each OTU was selected as the representative sequence. Representative sequences were again used to detect and discard putative chimeric OTUs using UCHIME.

In order to assign taxonomically the reads, we created a database of COI reference sequences by first selecting all GenBank entries (release 211), accessed on 06.01.2016[16]; with the query "COI AND 500:10000[Sequence Length]". Reference sequences were kept if they had taxonomic information at family, genus and species ranks and if they did not contain any ambiguous nucleotide. Ambiguous species annotations (e.g. sp., cf., aff., nr., n.sp., pr.) were all normalized to "sp.". Only one sequence was conserved for each unique taxonomic path (including 16 labelled ranks from superkingdom to species). The final reference database contained 425,824 sequences, of which 217,544 were Insecta (763 families, 14,857 genera). Then, all de-replicated reads were taxonomically assigned using the naïve Bayesian classifier[17] at a consensus threshold of 60%. The OTUs were finally assigned to the longest taxonomic path shared by at least 60% of their reads. As most GenBank COI sequences were produced with the same primer pair as used in our study and as end-gaps have no effect on the naïve Bayesian classifier assignments, it was not necessary to cut our reference database to the amplified region.

Step 3: *clean-up.* Singleton OTUs (N=395), which have a high probability of originating from sequencing errors, were removed from the data set. Non-target taxa OTUs (N=38; bacteria, fungi, unclassified eukaryotes, Mollusca, Nematoda, Arachnida) were also excluded. The

remaining 592 OTUs, representing insects (orders: Diptera, Lepidoptera, Coleoptera and Hymenoptera; Supplementary Figure 4, Supplementary Dataset), and accounting for a total of 84,911 sequence reads, were used for further statistical analysis. The number of total reads per site is shown in Supplementary table 12. The number of reads was not correlated with OTU richness across our dataset (Pearson's product-moment correlation r=0.033, N=18, P>0.05), suggesting we had sufficient reads to saturate our OTU assessment per site (Supplementary Figure 6). Detected number of OTUs, rarefied OTU richness and extrapolated total OTU richness (Chao 1) were highly correlated (P<0.001, Supplementary Table 13)

To verify independently our *de novo* clustering using cd-hit, we also used VSEARCH[18], a 56-bit reimplementation of the well know 32-bit USEARCH-UCLUST[19], to cluster and assign quality filtered and de-replicated reads. VSEARCH generated a larger number of OTUs (655 compared to 592 with UCHIME). However, there was a high correlation between the number of OTUs generated by the two methods across our 18 sites (Pearson's r=0.99; P<0.001). This suggests that our pipeline for *de novo* clustering and assignment of insect OTUs was robust. Pipeline scrips are available in a figshare Digital Repository [https://doi.org/10.6084/m9.figshare.10304795.v1].

Most of the 592 flying insect OTUs could be assigned to a species; Coleoptera: 53 OTUs, 12 of which were not assigned to species; Diptera: 342 OTUs, 225 of which were not assigned to species; Hymenoptera: 116 OTUs, 31 of which were not assigned to species; Lepidoptera: 81 OTUs, 16 of which were not assigned to species. For the well sampled bee species of Germany, we could assign species names to 40 of 46 OTUs and assign the remaining 6 OTUs to a unique taxon within a genus, which is consistent with public databases (e.g. NCBI) comprising 503 fully

‘compliant’ barcoded bee species of the 571 currently recognised German species [20]. Species names were checked against faunal lists to confirm their presence in the study region (Coleoptera: expert opinion, Matthias Seidel; Diptera: expert opinion, Martin Musche; Hymenoptera: expert opinion, co-author Paxton; Lepidoptera: expert opinion, co-author Settele). An OTU table with species names is available in a figshare Digital Repository [https://doi.org/10.6084/m9.figshare.10304795.v1].

To test the robustness of our pipeline, we used the raw sequence data (Sanger and *454*) from seven mock arthropod community datasets provided by Yu et al.[4]. With our pipeline applied to Yu et al.’s [4] 454 pyrosequencing dataset, we were able to recover a large proportion (88.5%) of the original Sanger sequenced taxonomic information (Supplementary Table 14). This suggests that our bioinformatics pipeline was well able to capture the diversity of flying insect species in a 454-NGS dataset and was a good compromise between over- and under-splitting.

Supplementary References

1. Theodorou, P. *et al.* The structure of flower visitor networks in relation to pollination across an agricultural to urban gradient. *Funct. Ecol.* **31**, 838–847 (2017).

2. Theodorou, P. *et al.* Pollination services enhanced with urbanization despite increasing pollinator parasitism. *Proc. Biol. Sci.* **283**, 20160561 (2016).

3. Hebert, P. D. N., Cywinska, A., Ball, S. L. & deWaard, J. R. Biological identifications through DNA barcodes. *Proc. R. Soc. London B Biol. Sci.* **270**, (2003).

4. Yu, D. W. *et al.* Biodiversity soup: metabarcoding of arthropods for rapid biodiversity assessment and biomonitoring. *Methods Ecol. Evol.* **3**, 613–623 (2012).

5. Ji, Y. *et al.* Reliable, verifiable and efficient monitoring of biodiversity via metabarcoding. *Ecol. Lett.* **16**, 1245–1257 (2013).

6. Shokralla, S. *et al.* Massively parallel multiplex DNA sequencing for specimen identification using an Illumina MiSeq platform. *Sci. Rep.* **5**, 9687 (2015).

7. Folmer, O., Black, M., Hoeh, W., Lutz, R. & Vrijenhoek, R. DNA primers for

amplification of mitochondrial cytochrome c oxidase subunit I from diverse metazoan invertebrates. *Mol. Mar. Biol. Biotechnol.* **3**, 294–9 (1994).

8. Geiger, M. *et al.* Testing the Global Malaise Trap Program – How well does the current barcode reference library identify flying insects in Germany? *Biodivers. Data J.* (2016). doi:10.3897/BDJ.4.e10671

9. Radzevičiūtė, R. *et al.* Replication of honey bee-associated RNA viruses across multiple bee species in apple orchards of Georgia, Germany and Kyrgyzstan. *J. Invertebr. Pathol.* **146**, 14–23 (2017).

10. Gaspar, J. M. & Thomas, W. K. FlowClus: efficiently filtering and denoising pyrosequenced amplicons. *BMC Bioinformatics* **16**, (2015).

11. Martin, M. Cutadapt removes adapter sequences from high-throughput sequencing reads. *EMBnet.journal* **17**, 10 (2011).

12. Quince, C., Lanzen, A., Davenport, R. J. & Turnbaugh, P. J. Removing noise from pyrosequenced amplicons. *BMC Bioinformatics* **12**, (2011).

13. Edgar, R. C., Haas, B. J., Clemente, J. C., Quince, C. & Knight, R. UCHIME improves sensitivity and speed of chimera detection. *Bioinformatics* **27**, 2194–200 (2011).

14. Schloss, P. D. *et al.* Introducing mothur: open-source, platform-independent, community-supported software for describing and comparing microbial communities. *Appl. Environ. Microbiol.* **75**, 7537–41 (2009).

15. Fu, L., Niu, B., Zhu, Z., Wu, S. & Li, W. CD-HIT: accelerated for clustering the next-generation sequencing data. *Bioinformatics* **28**, 3150–2 (2012).

16. Benson, D. A. *et al.* GenBank. *Nucleic Acids Res.* **41**, D36-42 (2013).

17. Wang, Q., Garrity, G. M., Tiedje, J. M. & Cole, J. R. Naive Bayesian classifier for rapid assignment of rRNA sequences into the new bacterial taxonomy. *Appl. Environ. Microbiol.* **73**, 5261–7 (2007).

18. Rognes, T., Flouri, T., Nichols, B., Quince, C. & Mahé, F. VSEARCH: a versatile open source tool for metagenomics. *PeerJ* (2016). doi:10.7717/peerj.2584

19. Edgar, R. C. Search and clustering orders of magnitude faster than BLAST. *Bioinformatics* (2010). doi:10.1093/bioinformatics/btq461

20. Schmidt, S., Schmid-Egger, C., Morinière, J., Haszprunar, G. & Hebert, P. D. N. DNA barcoding largely supports 250 years of classical taxonomy: identifications for Central European bees (Hymenoptera, Apoidea). *Mol. Ecol. Resour.* **15**, 985–1000 (2015).
