# Supplementary material for: Urban areas as hotspots for bees and pollination but not a panacea for all insects
Source: Nat Commun. 2020 Jan 29;11:576. doi: 10.1038/s41467-020-14496-6 (PMC6989530; doi:10.1038/s41467-020-14496-6)
Supplement: Supplementary file 3 — Reporting Summary [file 41467_2020_14496_MOESM3_ESM.pdf]

nature research

Corresponding author(s): Theodorou, Panagiotis

Last updated by author(s): Dec 24, 2019

Reporting Summary

Nature Research wishes to improve the reproducibility of the work that we publish. This form provides structure for consistency and transparency in reporting. For further information on Nature Research policies, see Authors & Referees and the Editorial Policy Checklist .

Statistics

For all statistical analyses, confirm that the following items are present in the figure legend, table legend, main text, or Methods section.

n/a	Confirmed	
☐	☒	The exact sample size (*n*) for each experimental group/condition, given as a discrete number and unit of measurement
☐	☒	A statement on whether measurements were taken from distinct samples or whether the same sample was measured repeatedly
☐	☒	The statistical test(s) used AND whether they are one- or two-sided<br>*Only common tests should be described solely by name; describe more complex techniques in the Methods section.*
☐	☒	A description of all covariates tested
☐	☒	A description of any assumptions or corrections, such as tests of normality and adjustment for multiple comparisons
☐	☒	A full description of the statistical parameters including central tendency (e.g. means) or other basic estimates (e.g. regression coefficient) AND variation (e.g. standard deviation) or associated estimates of uncertainty (e.g. confidence intervals)
☐	☒	For null hypothesis testing, the test statistic (e.g. *F*, *t*, *r*) with confidence intervals, effect sizes, degrees of freedom and *P* value noted<br>*Give P values as exact values whenever suitable.*
☒	☐	For Bayesian analysis, information on the choice of priors and Markov chain Monte Carlo settings
☐	☒	For hierarchical and complex designs, identification of the appropriate level for tests and full reporting of outcomes
☐	☒	Estimates of effect sizes (e.g. Cohen's *d*, Pearson's *r*), indicating how they were calculated

Our web collection on statistics for biologists contains articles on many of the points above.

Software and code

Policy information about availability of computer code

Data collection	Data on landscape heterogeneity were obtained using Quantum GIS v2.18 (https://qgis.org) and land cover data obtained from Geofabrik GmbH (https://www.geofabrik.de/).
Data analysis	All statistical analyses were performed in R statistical software v 3.5.2 using the packages picante v. 1.7, vegan v.2.5-2 , MuMIn v. 1.42.1, lme4 v. 1.1-18-1, ape v. 5.1 and piecewiseSEM v. 2.0.2. Bioinformatics analysis scripts used will be available upon publication in figshare Digital Repository (https://figshare.com/).

For manuscripts utilizing custom algorithms or software that are central to the research but not yet described in published literature, software must be made available to editors/reviewers. We strongly encourage code deposition in a community repository (e.g. GitHub). See the Nature Research guidelines for submitting code & software for further information.

Data

Policy information about availability of data

All manuscripts must include a data availability statement. This statement should provide the following information, where applicable:

- Accession codes, unique identifiers, or web links for publicly available datasets
- A list of figures that have associated raw data
- A description of any restrictions on data availability

Demultiplexed, raw 454-pyrosequencing reads are available under the accession number SRP096003 at the NCBI Sequence Read Archive database (http://www.ncbi.nlm.nih.gov/Traces/sra). Metadata and the bioinformatics analysis script used for the metabarcoding are available upon publication in the Dryad Digital Repository (http://datadryad.org/).

Field-specific reporting

Please select the one below that is the best fit for your research. If you are not sure, read the appropriate sections before making your selection.

☐ Life sciences ☐ Behavioural & social sciences ☒ Ecological, evolutionary & environmental sciences

For a reference copy of the document with all sections, see nature.com/documents/nr-reporting-summary-flat.pdf

Ecological, evolutionary & environmental sciences study design

All studies must disclose on these points even when the disclosure is negative.

Study description	We used a paired study design at flower-rich sites in nine independent German cities and nine nearby, equivalent, flower-rich rural sites to test the impact of urbanization on pollinator biodiversity and the ecosystem service of pollination. We used metabarcoding to evaluate flying insect diversity. To quantify pollination, we potted greenhouse-raised, insect-pollinator dependent red clover plants. We employed pan-traps to sample insects and to compare the diversity of flying insects at urban with those at rural sites, we measured species richness of the four main orders of flying insect pollinators: Diptera, Lepidoptera, Coleoptera, and Hymenoptera. In addition, we monitored all insects visiting the flowers of the experimental red clover plants (10 plants in each site) for five hours at each site in order to estimate flower visitation rates. Each urban-rural site pair was visited at the same time and for a total of five consecutive warm, non-windy days between June and August 2014. To determine the main ecological correlates of insect biodiversity and pollination in both rural and urban flower-rich sites, we gathered a series of local (patch) and landscape-scale variables potentially related to insect pollinators and pollination. These were (1) local flowering plant richness and abundance using 10 randomly placed 1 square meter quadrats at each of our sampling sites, (2) the proportion of semi-natural cover (grassland, meadows and scrub vegetation), (3) the proportion of forest, (4) the extent of arable (=agricultural) cover, (5) the proportion of residential and (6) commercial/industrial land cover, (7) the extent of botanical gardens, public parks and allotments, (8) landscape diversity and (9) edge density, as total length of 'green cover' (semi-natural and forest cover, botanical gardens, public parks, and allotments) patch edges divided by the total area, and which represents a quantification of landscape configuration. Given our paired 'urban-rural' experimental design, the rationale in our statistical analyses was to use site pair as a random factor and to compare between ecosystem type (urban versus rural). We controlled for potentially confounding local and landscape factors, unless we specifically aimed to model their relationship to predictor variables: dimensions of biodiversity and pollination.
Research sample	Our research sample is the insect community (Diptera, Lepidoptera, Coleoptera, and Hymenoptera) samples in each of our sites. It is characterized by overall insect biomass, species number and phylogenetic diversity.
Sampling strategy	We determined adequate sample size in our preliminary study, which has already been published (Theodorou et al. 2016) and from a published study of another group (Baldock et al. 2015).<br>Theodorou, P. et al. Pollination services enhanced with urbanization despite increasing pollinator parasitism. Proc. Roy. Soc. Lond. B Biol. Sci. 283, 20160561 (2016).<br>Baldock, K. C. R. et al. Where is the UK's pollinator biodiversity? The importance of urban areas for flower-visiting insects. Proc. R. Soc. Lond. B Biol. Sci. 282, 20142849 (2015).
Data collection	Insects were sampled using three blue, three yellow and three white pan traps (diameter: 42 cm, height: 2.8 cm) mounted on a stick at vegetation height at each site. Each pan trap was 2/3 filled with unscented soapy water and emptied every day for a total of five consecutive warm, non-windy days between June and August 2014. Insects from traps were killed on-site using 95% ethanol and stored in a -20°C freezer. Insect samples from each site were washed, dried and weighed using a balance. For assessment of species richness, we used next generation sequencing (NGS)-based metabarcoding. All people involved are listed in acknowledgements.
Timing and spatial scale	Each urban-rural site pair was visited at the same time and for a total of five consecutive warm, non-windy days at one point between 12/6/2014 and 10/8/2014. We sampled insects in cities and rural locations in central and eastern Germany.
Data exclusions	No data were excluded from the analyses.
Reproducibility	We used a highly replicated and statistically robust experimental design across multiple, paired sites.
Randomization	All samples collected for our analyses were from well defined, pre-selected locations, and samples were collected from all locations. Thus our sampling design was 'fully crossed'.
Blinding	All analyses were performed blind. This is especially relevant for our experimental pollination data, in which we collected seed from open versus closed flowers, and for our next-generation-sequencing data, in which we meta-barcoded pan-trapped insects. For these two datasets, bags/tubes containing material were given a unique number code that did not contain details of treatment, then data were collected/generated, and only afterwards were treatments allocated to processed data.

Did the study involve field work? ☒ Yes ☐ No

Field work, collection and transport

Field conditions	Temperatures exceeded 16°C, wind speed was less than 2 m/s at 1 m above ground level, and skies were sunny (<50% cloud cover) on all sampling days. These conditions are optimal to sample insects in our region.

Location	To test the association between urban versus rural land use on insect pollinators and pollination, we used a well-replicated study design across nine major cities in central and eastern Germany. Cities were Berlin, Braunschweig, Chemnitz, Dresden, Göttingen, Halle, Jena, Leipzig and Potsdam. Urban Berlin (52.45289 N 13.31002 E), Rural Berlin (52.16986 N 13.48448 E), Urban Braunschweig (52.26870 N 10.53336 E), Rural Braunschweig (52.20853 N 11.11153 E), Urban Chemnitz (50.85040 N 12.89103 E), Rural Chemnitz (50.96313 N 13.08918 E), Urban Dresden (51.04314 N 13.75754 E), Rural Dresden (50.94165 N 13.43837 E), Urban Göttingen (51.53826 N 09.93850 E), Rural Göttingen (51.54377 N 10.38625 E), Urban Halle (51.48966 N 11.96135 E), Rural Halle (51.39112 N 11.87891 E), Urban Jena (50.93119 N 11.58430 E), Rural Jena (50.82824 N 11.30146 E), Urban Leipzig (51.32920 N 12.39198 E), Rural Leipzig (51.18594 N 12.49890 E), Urban Potsdam (52.40796 N 13.02213 E) and Rural Potsdam (52.28192 N 12.83659 E).
Access and import/export	Fieldwork permits were issued by the responsible state environmental offices of Saalekreis (RL-0387), Stadt Halle (RL-0387), Leipzig (364.620/25/18/2), Stadt Leipzig (36.11-36.45.12/4/14-10-MH), Weimarer Land (III/UA/Schlo/Befreiung/17/01), Stadt Jena (enehmig_Befreiung\2014\Ausnahmegenehmigung\Fang+Besitz/AV10_AG06_14.odt), Mittelsachsen (23.4-55410704_Theodorou_2014), Stadt Dresden (86.44-12-0299/20937), Mittelsachsen (23.4-55410704_Theodorou_2014), Stadt Chemnitz (488-3667), Börde (RL-0387),Stadt Braunschweig (61.41/2-11.8-3), Teltow-Flaming (LUGV_RS7-4743/83 +5#216113/2014), Potsdam-Mittelmark (LUGV_RS7-4743/83+5#216113/2014), Stadt Potsdam (LUGV_RS7-4743/83 +5#216113/2014), Stadt Göttingen (67.2.5 Wei). For Stadt Berlin and Eichsfeld we thank Mario Hildebrandt, Bernd Lange (both Stadt Berlin) and Achim Gagalik (Eichsfeld). Access to private property was given by the land owner. Access to Botanical Gardens were given by their Directors.
Disturbance	No disturbance was caused during our sampling.

Reporting for specific materials, systems and methods

We require information from authors about some types of materials, experimental systems and methods used in many studies. Here, indicate whether each material, system or method listed is relevant to your study. If you are not sure if a list item applies to your research, read the appropriate section before selecting a response.

Materials & experimental systems

n/a	Involved in the study
☒	☐ Antibodies
☒	☐ Eukaryotic cell lines
☒	☐ Palaeontology
☐	☒ Animals and other organisms
☒	☐ Human research participants
☒	☐ Clinical data

Methods

n/a	Involved in the study
☒	☐ ChIP-seq
☒	☐ Flow cytometry
☒	☐ MRI-based neuroimaging

Animals and other organisms

Policy information about studies involving animals; ARRIVE guidelines recommended for reporting animal research

Laboratory animals	No laboratory animals were involved in the study.
Wild animals	For the purpose of our study, we collected flying insects (Diptera, Lepidoptera, Coleoptera and Hymenoptera) using pan-traps in the field, which were killed on-site in 95% ethanol.
Field-collected samples	Samples in 95% ethanol were stored in a -20°C freezer, except for short time periods during transport.
Ethics oversight	No ethical approval was required.

Note that full information on the approval of the study protocol must also be provided in the manuscript.
