# Supplementary material for: Urban areas as hotspots for bees and pollination but not a panacea for all insects
Source: Nat Commun. 2020 Jan 29;11:576. doi: 10.1038/s41467-020-14496-6 (PMC6989530; doi:10.1038/s41467-020-14496-6)
Supplement: Supplementary file 4 — Description of Additional Supplementary Files [file 41467_2020_14496_MOESM4_ESM.pdf]

Description of Additional Supplementary Files

File Name: Supplementary Data 1
Description: Operational Taxonomic Units Dataset
